# RA-map: building a state-of-the-art interactive knowledge base for rheumatoid arthritis

**DOI:** 10.1093/database/baaa017

**Published:** 2020-04-20

**Authors:** Vidisha Singh, George D Kalliolias, Marek Ostaszewski, Maëva Veyssiere, Eleftherios Pilalis, Piotr Gawron, Alexander Mazein, Eric Bonnet, Elisabeth Petit-Teixeira, Anna Niarakis

**Affiliations:** 1 Laboratoire Européen de Recherche pour la Polyarthrite Rhumatoïde - Genhotel, Univ Evry, Université Paris-Saclay, 2, rue Gaston Crémieux, 91057 EVRY-GENOPOLE cedex, Evry, France; 2 Arthritis and Tissue Degeneration Program, Hospital for Special Surgery, 535 East 70th Street, New York, NY 10021, USA; 3 Weill Cornell Medical Center, Weill Department of Medicine, 525 East 68th Street, New York, NY 10065, USA; 4 Luxembourg Centre for Systems Biomedicine, University of Luxembourg, 6 Avenue du Swing, L-4367 Belvaux, Luxembourg; 5 eNIOS Applications P.C., R&D department, Alexandrou Pantou 25, 17671, Kallithea-Athens, Greece; 6 Centre National de Recherche en Génomique Humaine (CNRGH), CEA, 2 rue Gaston Crémieux, CP5706 91057 EVRY-GENOPOLE cedex, Evry, France

## Abstract

Rheumatoid arthritis (RA) is a progressive, inflammatory autoimmune disease of unknown aetiology. The complex mechanism of aetiopathogenesis, progress and chronicity of the disease involves genetic, epigenetic and environmental factors. To understand the molecular mechanisms underlying disease phenotypes, one has to place implicated factors in their functional context. However, integration and organization of such data in a systematic manner remains a challenging task. Molecular maps are widely used in biology to provide a useful and intuitive way of depicting a variety of biological processes and disease mechanisms. Recent large-scale collaborative efforts such as the Disease Maps Project demonstrate the utility of such maps as versatile tools to organize and formalize disease-specific knowledge in a comprehensive way, both human and machine-readable. We present a systematic effort to construct a fully annotated, expert validated, state-of-the-art knowledge base for RA in the form of a molecular map. The RA map illustrates molecular and signalling pathways implicated in the disease. Signal transduction is depicted from receptors to the nucleus using the Systems Biology Graphical Notation (SBGN) standard representation. High-quality manual curation, use of only human-specific studies and focus on small-scale experiments aim to limit false positives in the map. The state-of-the-art molecular map for RA, using information from 353 peer-reviewed scientific publications, comprises 506 species, 446 reactions and 8 phenotypes. The species in the map are classified to 303 proteins, 61 complexes, 106 genes, 106 RNA entities, 2 ions and 7 simple molecules. The RA map is available online at ramap.elixir-luxembourg.org as an open-access knowledge base allowing for easy navigation and search of molecular pathways implicated in the disease. Furthermore, the RA map can serve as a template for *omics* data visualization.

## Introduction

Rheumatoid arthritis (RA) is a progressive inflammatory and autoimmune disease with unknown aetiology. It affects 0.5–1% of the world population, and disease characteristics involve synovial inflammation and hyperplasia, cartilage and bone destruction, production of autoantibodies like rheumatoid factor (RF) and anti-citrullinated protein (ACPA), and various systemic features such as cardiovascular, pulmonary, psychological and skeletal disorders (1). The pathogenesis of RA is a multistep process involving an intricate interplay between genetic, environmental and epigenetic mechanisms, a variety of intertwined signalling cascades and the expression of pro-inflammatory mediators (1, 2).

Systems Biology allows deciphering complex disease mechanisms by treating biological processes in living organisms as coordinated and interdependent events. Especially in human diseases, genes and proteins rarely act alone when affecting implicated cells, tissues or organs. To understand the molecular mechanisms underlying these phenotypes, one has to place the implicated biomolecules in their functional context and interconnect them. This way, a graphical representation of disease mechanisms is established and can be refined, validated and interpreted using the wealth of high-throughput biological data. Nevertheless, integration and organization of both graph and data in a systematic and standardized manner remains a challenge.

Molecular maps are widely used in biology to provide a useful and intuitive way of depicting a variety of biological processes and disease mechanisms. Examples of such maps include the gastrin and cholecystokinin receptor signalling (3), yeast stress response pathways (4), FceRI receptor signalling in allergy (5), mitogen-activated protein kinase (MAPK) pathways (6), Parkinson’s disease (7), Alzheimer’s disease (8), influenza A virus (9), asthma (10), cancer (11) and RA (12). Recent large-scale collaborative efforts such as the Disease Maps Project (13, 14), demonstrate the utility of such maps as versatile tools to organize and formalize disease-specific knowledge in a comprehensive way, both human and machine-readable.

In this work, we present a systematic effort to construct a fully annotated, expert validated, state-of-the-art knowledge base for RA in the form of a molecular map. The RA map illustrates molecular and signalling pathways implicated in the disease. Signal transduction is depicted from receptors to the nucleus in a systematic fashion using the Systems Biology Graphical Notation (SBGN) standard representation (15). High-quality manual curation, use of only human-specific studies and focus on small-scale experiments aim to limit false positives in the map. The RA map serves as an interactive knowledge base but also as a template for *omic* data visualization. *Omic* datasets can be superimposed on the map, pinpointing affected areas in different samples.

**Figure 1:**
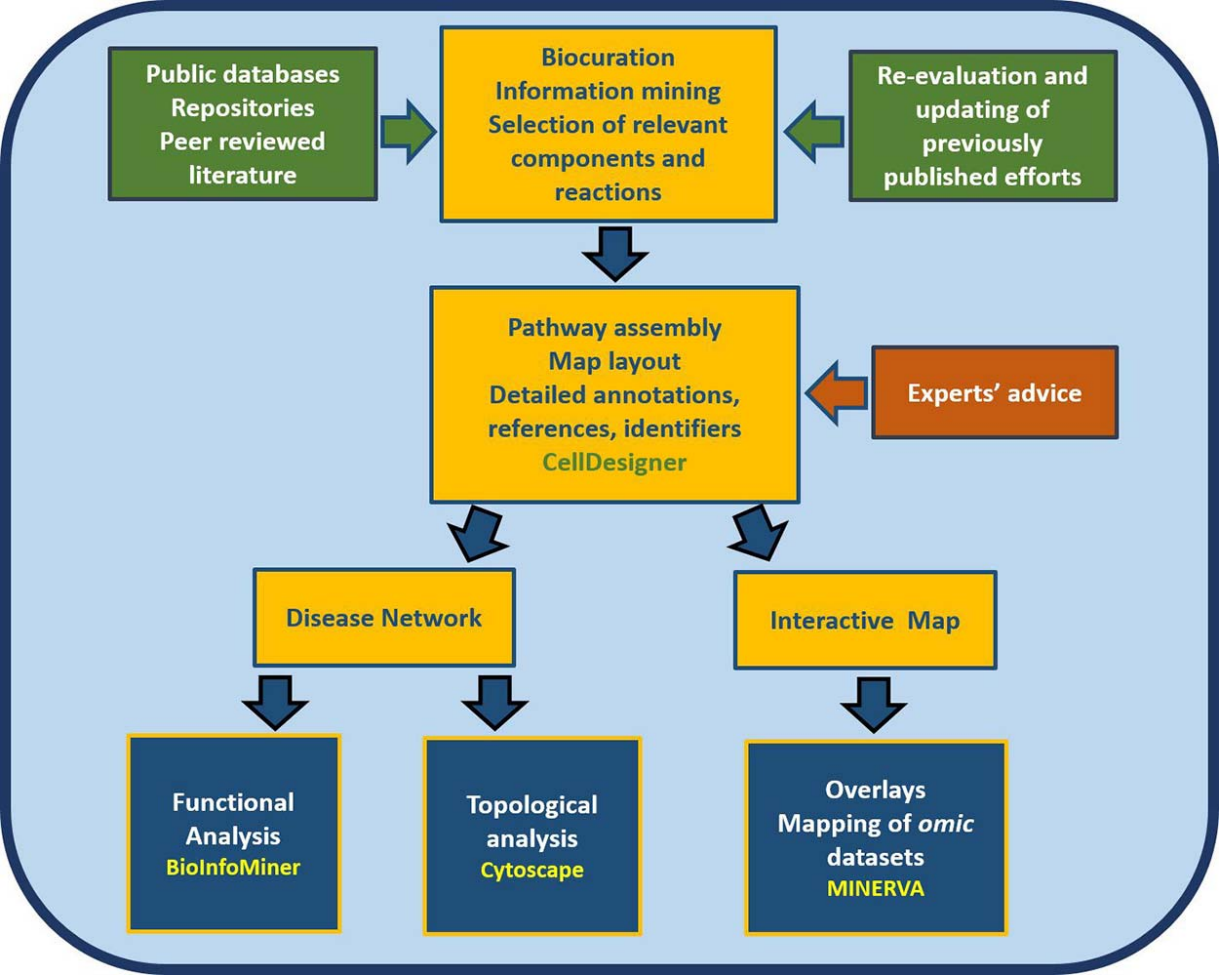
**Workflow for the construction and use of the RA map.** The assembly of the signalling and molecular pathways implicated in RA involves exhaustive manual curation and information mining from literature, public databases and repositories and the use of the software CellDesigner (18). The RA map contains mechanisms reported in the most recently published studies, after validation from RA experts. The map can be transformed into an online interactive knowledge base using the platform MINERVA (19). Functional enrichment and topological analysis is possible using the software BioInfoMiner (16) (https://bioinfominer.com) and Cytoscape (17), respectively.

Furthermore, the map is a good starting point for the development of a computational model, providing an intermediate step between a conceptual, mechanistic graph and an executable mathematical model (12). The article comprises three parts. In the first part, we present the process of constructing the RA map, highlighting the most critical pathways. In the second part, we transform the RA map into a state-of-the-art interactive knowledge base for the disease, which interfaces with various databases for content annotation and enrichment analysis of experimental results. In the third part, we use bioinformatics tools such as BioInfoMiner (16) (https://bioinfominer.com) and Cytoscape (17) for the analysis of the RA map as a complex biological network, revealing topological and functional aspects of the map (Figure 1).

## Methods

### Construction of the RA map

CellDesigner (18) is a structured diagram editor for the creation of gene-regulatory and biochemical networks. Networks are drawn using the Process Description visual language of SBGN, and are stored using the Systems Biology Markup Language (SBML) (20), a standard for representing models of biochemical and gene-regulatory networks. In a CellDesigner diagram, nodes represent species like proteins, genes, complexes and other molecules, and the edges denote the interaction between the nodes, which can be activation, inhibition, catalysis and state transition among other possible interactions (21, 22). A comprehensive molecular interaction map for RA was published in 2010 (23) with information derived from high-throughput data combined with interaction data from the KEGG pathway database (24–27) (http://www.genome.jp/kegg/pathway.html). The researchers of this study used 28 published studies for the construction of the first RA map that included experiments performed in different cell types/tissues/fluids such as the peripheral blood mononuclear cells, synovial fibroblasts, macrophages, chondrocytes, synovial tissues, bone, blood, and synovial fluid (Figure S1, Table S1). We used this RA map as a basis and extended it to create a state of the art map for RA. However, apart from updates, the first map has been significantly modified. A systematic effort was made to create an SBGN-compliant map, the first to our knowledge. We also removed from the map many factors and reactions that were either not disease-specific or did not follow the curation criteria (discussed in section Annotation and curation criteria). The map was restructured to depict a cell layout. We grouped the receptors by category (growth factors, cytokines, chemokines, integrins and Toll-like receptors). For the updating, keywords like ‘rheumatoid arthritis’, ‘pathogenesis of rheumatoid arthritis’, ‘cytokines involved in rheumatoid arthritis’, ‘factors involved in rheumatoid arthritis’, ‘signalling pathways in rheumatoid arthritis’ were used to select relevant literature after 2010 (or older than 2010 that would correspond to small-scale experiments, in order to annotate nodes and reactions already present in the map) with emphasis given on recent review articles and their reference lists. We added proteins, genes and cellular phenotypes to the map and used databases like KEGG pathway (24–27) (http://www.genome.jp/kegg/pathway.html), and Ingenuity Pathway Analysis (IPA) (28) to retrieve connections among them, where it was not possible to retrieve the links directly from the corresponding articles. All added factors were discussed thoroughly with RA experts before addition to the map and advice was taken for the best possible representation of their mechanism of action.

### Annotation and curation criteria

We carried out an exhaustive literature search for new proteins, genes and other molecules involved in the pathogenesis of RA. Relevant keywords and key phrases like ‘Pathogenesis of RA’, ‘Cytokines in the pathogenesis of RA’, ‘Therapeutic targets in RA’ among many others were used to filter the literature abstracts and studies in PubMed and Google Scholar. Along with it, we used peer reviewed articles concerning RA and searched their bibliographies to mine relevant information. We focused only on studies based on cells, fluids and tissues of human origin using small-scale experiments, in an attempt to limit false positives from gene expression data used to construct the first RA map. New RA mediators were added and referenced with at least two PubMed IDs. However, we made some exceptions during the building of the map. For molecules that were either published very recently (since January 2018) or were part of well-characterized pathways involved in RA, we used one PubMed or KEGG ID. For the purposes of this project, we aimed to be inclusive of the whole spectrum of RA. In this context, we used RA as a defining criterion and did not make the distinction between sero-negative and sero-positive RA when reviewing the literature.

We added annotations for all the components (proteins, RNAs and genes) and reactions present in the CellDesigner XML file using the sections text NOTE and Minimal Information Requested In the Annotation of Models (MIRIAM) (29), which are human and machine-readable formats respectively (Figure S2). In the MIRIAM segment, we added PubMed IDs for different cell types with the tag ‘bqbiol: is described by’. In the NOTE section, we added text information about KEGG pathway identifiers used to cross-validate interactions.

### Evaluation of components and reactions

We carefully evaluated all elements and reactions of the previous RA map and added annotations concerning experimental validation with small-scale experiments where possible. Molecules, for which we could not find small-scale experiments, were kept if appeared in at least two high-throughput studies. We removed from the map molecules that failed to fulfil the above criteria.

### Compartments, structure and layout

To improve the layout of the molecular map, we used the CellDesigner plugin Relayout Model (http://www.celldesigner.org/plugins.html). The RA map includes six compartments, namely extracellular space, plasma membrane, cytoplasm (including Golgi apparatus, endoplasmic reticulum, and mitochondria), nucleus, secreted molecules and cellular phenotypes.

A cellular phenotype can be viewed as the endpoint of multiple cellular processes that define and shape the morphology and function of the cell, dictating its fate. Extracellular space includes the protein ligands outside the cell that can form a complex with the plasma membrane receptors and proteins resulting in the activation of several signalling cascades. Cytoplasm compartment includes the signalling proteins, enzymes, small molecules and transcription factors, which are subsequently transported to the nucleus and are involved in gene expression regulation. The nucleus compartment includes transcription factors transported from the cytoplasm, genes and RNAs (miRNA and mRNA). A separate compartment contains proteins secreted out of the cell and, finally, a dedicated compartment contains cellular phenotypes relevant for RA. The RA map has the form of a cell with surrounding extracellular space, the cytoplasmic area containing organelles, proteins and small molecules, the nucleus with gene-regulatory mechanisms, secreted molecules and cellular phenotypes. We used a distinct colour code for the components in the RA map: plasma membrane receptors in peach, proteins in purple, genes in green, RNAs in red and cellular phenotypes in yellow. Inhibition edges are represented in red colour, while for all others like state transition, catalysis, transport, reduced physical stimulation and heterodimer association we used black colour.

### Experts’ advice and feedback

Experts’ curation is critical to reconstructing molecular and cellular interactions from the available literature. Due to the complexity of RA regarding cell types (macrophages, lymphocytes, endothelial cells, synovial fibroblasts), mediators of inflammation (cytokines, chemokines, growth factors, tissue-degrading enzymes) and the variety of biological processes implicated in the disease, the review of the map by RA experts was necessary for an accurate representation of disease hallmarks. To provide a systematic and comprehensive molecular map, we used SBGN standards and a cell layout. We took advice from experienced scientists in both biological and computational domains to make the content comprehensive and functional for different types of users such as experimental biologists, clinicians, computational modellers and bioinformaticians. The RA map layout, the representation of various levels of information and the validity of molecules and pathways included in the RA map, were carefully examined in this context.

### SBGN standards and process description map validation

The SBGN (15) is a standard for the visual representation of biological/biochemical processes as networks. Three types of SBGN languages cover different ways to represent biological networks, Process Description (PD), Entity-Relationship (ER) and Activity Flow (AF) (30). The RA map is a PD map showing the detailed biological processes implicated in RA. We systematically checked the compliance to the SBGN standard. For keeping the diagram compact and avoid repeating the same pattern multiple times (activation of protein production from an empty set), we used the translation connectors. VANTED (Visualisation and Analysis of Networks containing Experimental Data) (31), is a framework for systems biology applications with functionalities ranging from network reconstruction, data visualization, integration of various data types to network simulation using systems biology standards for visualization and data exchange. We used SBGN-ED (an add-on for VANTED for editing, validating and translating of SBGN maps) (32) to validate our SBGN PD encoding of the RA map. As this tool works with SBGN-ML file format, we utilized the CellDesigner to SBGN converter (https://royludo.github.io/cd2sbgnml) for converting the CellDesigner XML file into SBGN-ML format and subsequently import the file to VANTED for further analysis.

#### Web-based MINERVA map

The RA map is available as an online interactive map using MINERVA (Molecular Interaction NEtwoRks VisuAlization) platform (19). MINERVA is a web service that supports curation, annotation and visualization of molecular interaction networks in the SBGN-compliant format. MINERVA provides automated content annotation and verification, along with mapping of drug targets and overlaying experimental data on the visualized networks. Automated annotations (HGCN) and curator’s annotations for every component and reaction are displayed in the left panel (see Figure 3A). The user can also visualize cell-specific data based on curated overlays or analyse patients’ *omic* datasets (see Figure 8). Moreover, MINERVA provides an interface for interrogating several other databases such as DrugBank (33) (https://www.drugbank.ca/), CHEMBL (34) (https://www.ebi.ac.uk/chembl/), CTD (35) (http://ctdbase.org) and miRTarBase (36) (http://mirtarbase.mbc.nctu.edu.tw).

#### Overlays

We provide three different types of overlays with the RA map. The first type corresponds to cell, tissue and fluid specific overlays. The RA map is a global map, integrating data and information from various sources. As a result, it has reactions and components that come from different cell or tissue types. We have grouped the sources into seven distinct groups that we provide as overlays. The groups are synovial fibroblasts, synovial tissue, peripheral blood mononuclear cells, blood, synovial fluid, chondrocytes and macrophages (Table S1). These overlays allow visualizing cell or tissue-specific interactions and molecules. The second type of overlay comes from publicly available datasets and facilitates visualization of mapping components onto the RA map. The third type of overlays concerns canonical pathways retrieved from REACTOME, EBI for TNF, IL6, MAPK and Interferon signalling (Table S2).

#### BioInfoMiner analysis

The algorithm performs a topological analysis of semantic networks, derived from ontologies (Gene Ontology (37, 38), Human Phenotype Ontology (39) and Mammalian Phenotype Ontology (40)) and pathway databases with hierarchical structure, like REACTOME (41–43). It employs a graph-theoretical method that corrects the annotation bias of community ontologies, performs enrichment analysis to assess the over-representation of terms and ranks the related genes according to their connectivity in the corrected semantic network (44, 45). Systemic processes are clusters of terms that share maximum semantic similarity among them, but minimal similarity among other clusters. The highly ranked genes are those associated with many systemic processes, and thus, they are considered hub genes in the semantic network, assuring cross-talking among distinct, orthogonal (inter-independent) processes. Finally, the application derives a signature, consisting of the mapping of the prioritized genes to a minimal set of clustered systemic processes. Furthermore, BioInfoMiner provides a pharmacogenomic analysis, as the derived hub genes constitute putative drug targets.

### Topological and gene ontology enrichment analysis with Cytoscape

The RA map XML file was imported in Cytoscape, version 3.5.0, and the built-in NetworkAnalyzer function was used for topological analysis (17).

## Results

### A comprehensive molecular interaction map for RA

The RA map graphically illustrates signalling pathways, gene expression regulation, molecular mechanisms and cellular phenotypes involved in the pathogenesis of the disease. As shown in Figure 1, and explained in detail in the methodology section, the RA map requires exhaustive literature curation, information mining from relevant databases along with continuous updating and advice from domain experts. Importantly, the interactions shown in the diagram represent a graphical model encoded using a standardized format, making the map computationally tractable.

**Figure 2:**
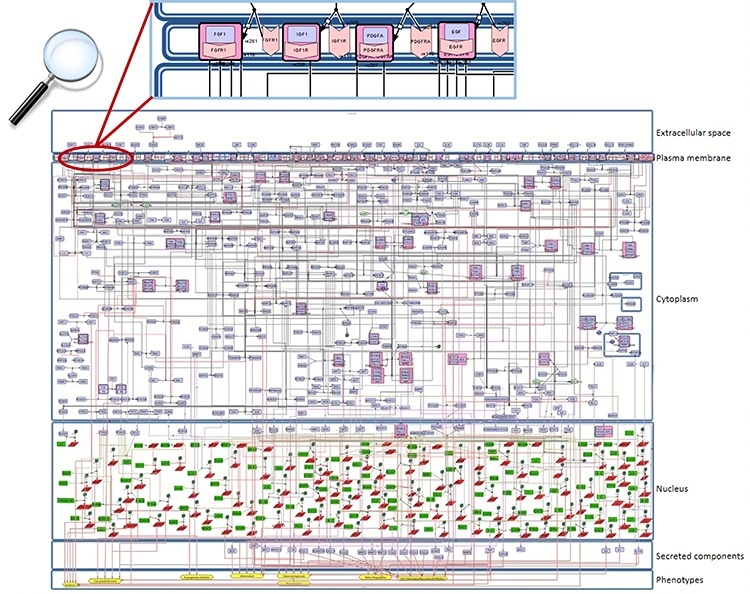
**Snapshot of the SBGN-compliant RA map.** The map is colour-coded with proteins in purple, genes in green, RNAs in red and phenotypes in yellow. State transitions and catalysis reactions are displayed in black, and the inhibitions are in red. Compartments are distinguished as bounding boxes. The map was built using CellDesigner, version 4.4 (18). Modifications to the SBGN format: translation arcs are used to keep the representation compact, as well as the gene and RNA shapes.

For the construction of the map, we used the graphical editor CellDesigner (18). In Figure 2, one can see an overview of the RA map. We constructed the RA map following the SBGN Process Description format (46). We made only one exception concerning the choice of the translation and transcription representation, for which we used the CellDesigner’s system of symbols. The RA Map features 506 species, 449 reactions and 8 cellular phenotypes. The biomolecules in the map are 303 proteins, 61 molecular complexes, 106 genes, 106 RNA entities, 2 ions and 7 simple chemical species like for example cAMP, H_2_O_2_ or PIP_3_. Proteins include extracellular, membrane and cytoplasmic proteins comprising signalling proteins, enzymes and transcription factors. The reactions are classified as state transitions, catalyses, inhibitions, transports, heterodimer associations, dissociations, Boolean AND gates and reduced physical stimulations. All the components in the map have at least two manually curated PubMed references, giving overall 353 publications covering a period from 1973 to 2019 (Figure S3).

The RA map is organized in the form of a cell representing the flow of information from the extracellular space (ligands) to the plasma membrane (ligand–receptor complexes) and then to the cytoplasm (signalling pathways), the nucleus (gene regulation) and the secreted compartment or cellular phenotypes (Figure 2).

### Molecular pathways covered in the RA map

The RA map contains hallmark cellular and molecular pathways that participate in disease pathogenesis. In signalling cascades, the activation occurs as a response to an upstream stimulus. After activation, the signal propagates through a series of coupled reactions from the plasma membrane to the cytoplasm, to regulate key factors that are responsible for gene regulation and different cellular phenotypes. The RA map includes the following upstream stimuli:

(i) Cytokines and chemokines: a diverse group of proteins like tumour necrosis factor (TNF) and interleukins to list a few, implicated in various phases of RA pathogenesis by promoting autoimmunity, initiating and maintaining chronic inflammatory synovitis and driving cartilage and bone destruction (47–49);
(ii) Growth factors: such as epidermal growth factor (EGF), fibroblast growth factor (FGF), insulin-like growth factor (IGF), vascular endothelial growth factor (VEGF), platelet-derived growth factor (PDGF), activate intracellular signalling pathways (such as PI3K-AKT pathway) and regulate a broad range of cellular functions like cell growth, survival, cell motility and apoptosis (50, 51);
(iii) Toll-like receptors (TLRs): TLR2 and TLR4 are primarily expressed in synovial fibroblasts and macrophages in human RA joints (52–54). Activation of TLR2 and TLR4 results in recruitment of adaptor molecules such as MyD88, IRAK, TRAF6 and TANK-binding kinase (TBK)-1 and leads to the activation of MAPKs and NF-κB and the increased expression of various pro-inflammatory and tissue-destructive mediators (such as TNF, IL-6, chemokines and MMPs) (55, 56).

The activation of these upstream components leads to the activation of downstream pathways that include:

(i) The JAK-STAT pathway: this is an effective target in RA therapy. Many cytokines, including IL-6 and TNF, which are validated therapeutic targets in RA, activate directly (for example IL-6) or indirectly (for example TNF) this pathway by phosphorylating JAK proteins. JAKs, in turn, phosphorylate STATs, which then dimerize and translocate to the nucleus and bind to regulatory elements of DNA modulating the expression of target genes (57, 58). Activation of JAK-STAT pathway also results in the activation of suppression of cytokine signalling (SOCS), which operates as a feedback inhibitory loop aiming to terminate excessive activation of JAK-STAT (59).
(ii) The NF-κB pathway: it is involved in inflammation, cell survival and proliferation. Activated NF-κB is detected in immune cells (such as macrophages and lymphocytes) as well as in stromal cells (such as FLS and endothelial cells) and stimulates the transcription of arthritogenic mediators like IL-1, TNF, RANKL, PTGS2 and IL-6 in RA synovium. TNF, IL-1 and RANKL are key upstream RA-relevant triggers of the activation of the NF-κBpathway (60).
(iii) The MAPK pathway: all the three classes of MAPKs, namely ERK, JNK and p38, are found to be expressed and activated in synovial tissue in RA. A series of cytokines including among others TNF, IL-1 and IL-6 activate ERK, JNK and p38 MAPK in synovial tissue with successive induction of proinflammatory mediators such as cytokines and tissue destructive enzymes (e.g. MMP-1 and MMP-13) (61, 62) Negative feedbacks are required to keep in check the constitutive activation of MAPK proteins in order to control the excessive prolonged expression of pro-inflammatory genes (61).
(iv) The PI3K-AKT pathway: growth factors like VEGF and FGF induce the PI3K-AKT pathway (50, 63–65). Activated cellular AKT regulates immune cells, and survival of synoviocytes and chondrocytes by phosphorylating several downstream signalling proteins modulating mTOR, BAD, FOXO3 and tumour protein-73 (TP-73) (63).

All signalling cascades end at specific cellular outcomes grouped in eight distinct phenotypes such as inflammation (1, 51, 66, 67), cell chemotaxis/recruitment/infiltration (68, 69), matrix degradation (66, 70–73), osteoclastogenesis (66, 74, 75) and bone erosion (1, 66, 76, 77), angiogenesis (51, 66, 78, 79), apoptosis (66, 80–83) and finally cell survival/growth/proliferation (51, 84–86).

### Transforming RA map into a state of the art knowledge base using MINERVA

**Figure 3:**
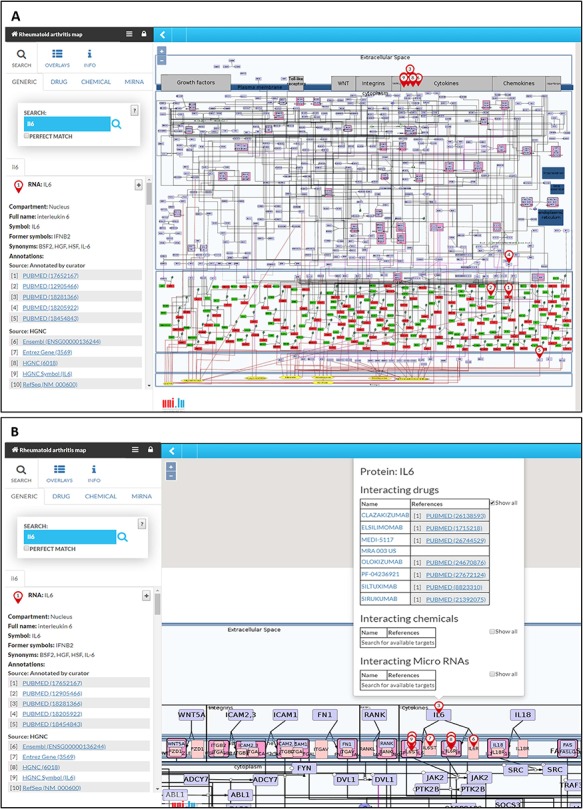
**The RA map in MINERVA platform.** (**A**) Users can use the search box to type in the element of interest. The resulting element shows up as pins on the map. Corresponding annotations of the searched element, like HGNC, Entrez Gene, RefSeq and Ensembl identifiers are displayed on the left panel along with the PubMed identifiers of the manually curated annotations. (**B**) Further clicking on the pin will display additional information about interacting drugs, chemicals and microRNAs for the element.

The RA map is available at ramap.elixir-luxembourg.org in the form of an interactive diagram, using the platform MINERVA (Molecular Interaction NEtwoRks VisuAlization) (Figure 3). Clicking on a biomolecule in the map, the user can choose to visualize interacting drugs, chemicals and miRNAs. The RA map interfaces with DrugBank (https://www.drugbank.ca/), CHEMBL (https://www.ebi.ac.uk/chembl/), CTD (http://ctdbase.org) and miRTarBase (http://mirtarbase.mbc.nctu.edu.tw).

**Figure 4:**
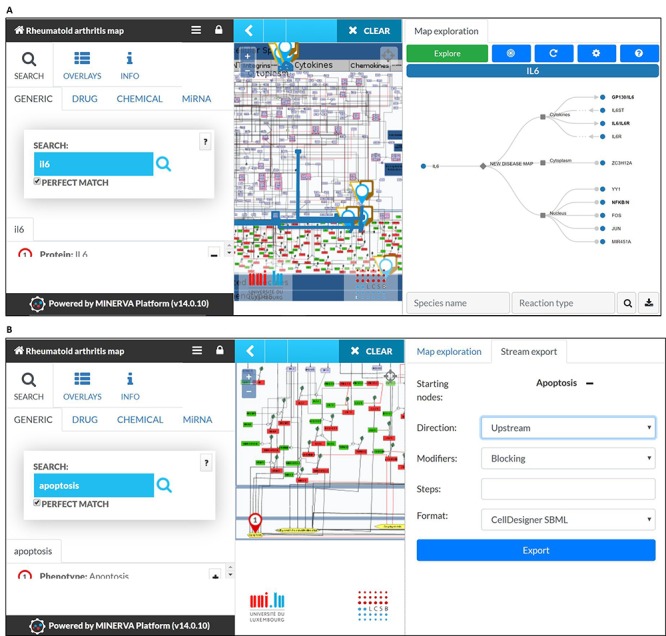
**MINERVA plugins.** (**A**) The tree plugin allows to navigate in dense networks by following interactions in a tree-like manner. (**B**) The stream plugin allows for downstream or upstream expansion when selecting a node of interest.

RA map offers custom visualization and export capabilities via MINERVA plugins (87). For instance, users can explore the RA map starting from a molecule of interest and easily follow its interactions, even throughout a dense and complex network. This functionality facilitates navigating through the contents and tracking the flow of the signal from the ligand to the corresponding phenotype (Figure 4A). Another feature of the RA map is the stream plugin, allowing for highlight and export of entire subnetworks in the map in one click. This feature is especially important to visualize the ensemble of signalling pathways converging on the same disease-related phenotype (Figure 4B).

### The RA map as a template for visualizing cell-specific overlays

**Figure 5:**
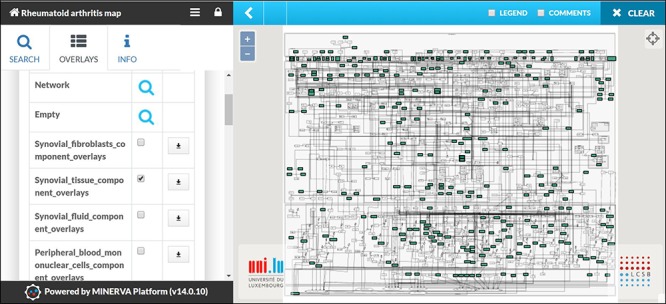
**Visualizing cell/tissue/fluid-specific parts of the RA map using dedicated overlays.** Snapshot of the visualization of the Synovial Tissue overlay.

The RA map contains information from various sources serving as a generic blueprint for disease mechanisms. However, due to extensive annotation and reference, the user can opt for visualizing cell-specific nodes and interactions. In the RA map, we have grouped our sources in seven distinct groups: synovial fibroblasts, synovial tissue, peripheral blood mononuclear cells (including PMNs), blood (including T and B cells), synovial fluid, chondrocytes and macrophages (Table S1). Synovial fibroblasts are the most frequent cell type in the RA map covering a total of 45%, followed by synovial tissue with 36% (Figure S1). In the RA map, the user can select to visualize one of the corresponding overlays, for example, synovial tissue overlay (Figure 5).

**Figure 6:**
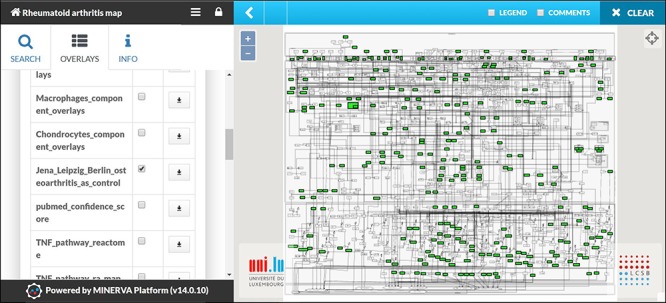
**Mapping of *Omic* datasets from RA synovial tissue.** The apoptosis and angiogenesis phenotypes appear to be inactive as no molecule leading to these cellular phenotypes is mapped.

**Figure 7:**
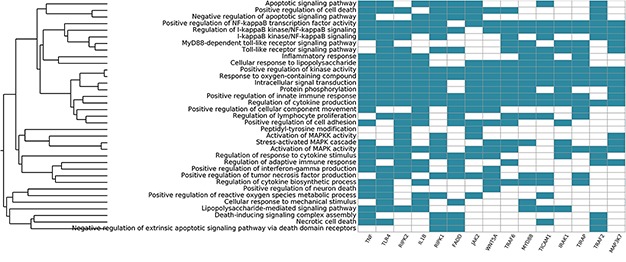
**Systemic functional analysis of the RA map using GO terms.** Heat map of the top 15 priority genes and their systemic interpretation using BioInfoMiner and GO terms.

**Figure 8:**
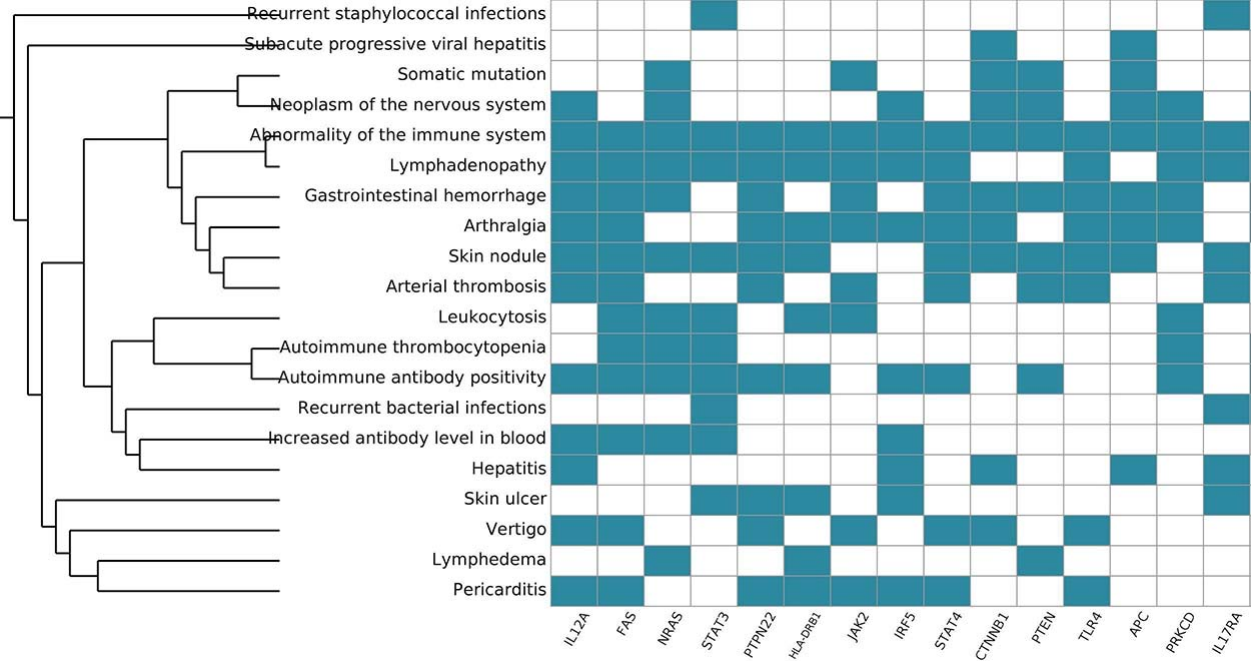
**Systemic functional analysis of the RA map using HPO terms.** Heat map of the top 15 priority genes and their systemic interpretation using BioInfoMiner and HPO terms.

**Figure 9:**
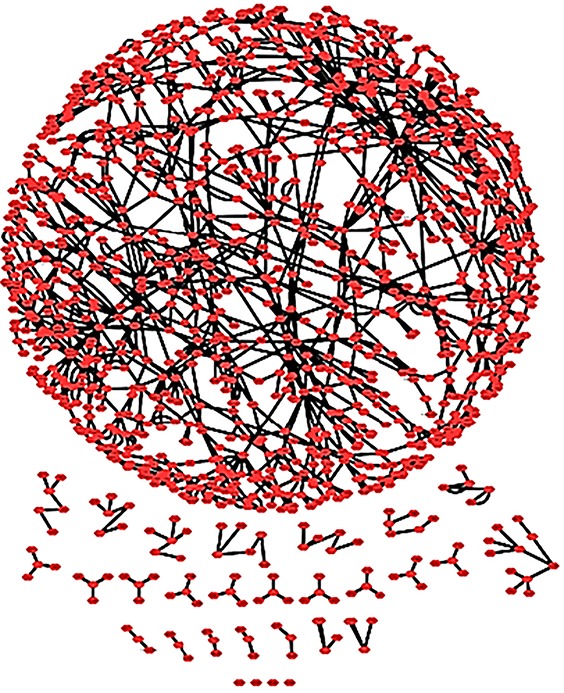
**The RA map as a complex network.** The RA network with spring embedded layout. One connected core and several smaller unconnected parts are shown.

**Figure 10:**
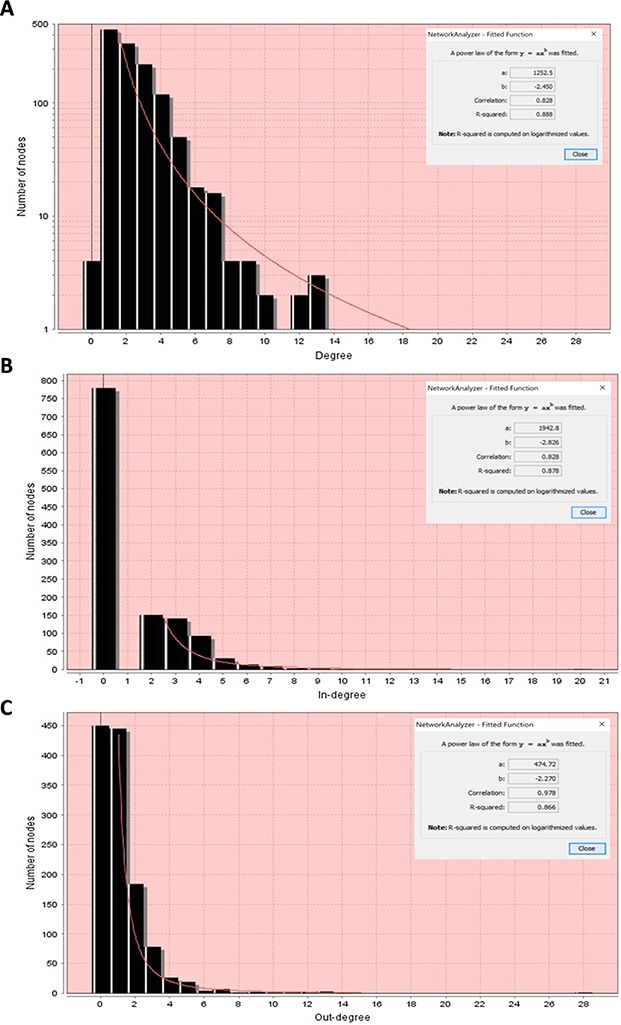
**Node degree distributions of the RA map with a fitted power law**. (**A**) Overall degree distribution. (**B**) In-degree distribution. (**C**) Out-degree distribution.

### Visualizing various datasets

We used publicly available datasets for visualization with the RA map. Our goal was to compare the differentially expressed pathways or map regions in different datasets. For this purpose, we used the datasets from transcriptomic data of synovial tissue (88). We performed differential expression analysis between Berlin, Leipzig and Jena datasets using osteoarthritis as control and visualized the mapping of 122 molecules to the RA map. Most pathways were highlighted, as molecules that lead to most phenotypes were present. Interestingly, we found enrichment for almost all cellular phenotypes except for apoptosis and angiogenesis. Molecules leading to six out of eight phenotypes were expressed, while molecules linked to the two mentioned phenotypes were absent (Figure 6).

### Systemic interpretation and pharmacogenomics analysis using BioInfoMiner

We also used the BioInfoMiner web application (16) (https://bioinfominer.com) to perform a functional analysis of the RA map. The application performs a biological interpretation of gene sets, which comprises detection and prioritization of systemic processes and pathways, as well as prioritization of genes based on their mapping to those processes. We used BioInfoMiner as a second layer of analysis to see if the functional enrichment would give results relevant to the autoimmune process and RA. We performed two sets of analyses using gene ontology (GO) and human phenotype ontology (PHO) terms. The first analysis using GO gave enrichment of terms like Inflammatory response, Regulation of cytokine production and Activation of MAPK activity, all relevant to pathways included in the RA map. The top five GO terms included apoptotic signalling pathway, positive regulation of cell death, negative regulation of apoptotic signalling pathway, positive regulation of NF-kappaB transcription factor activity and regulation of I-kappaB kinase/NF-kappaB signalling. It also gave a list of 48 prioritized genes (Table S3). The top 10 priority genes obtained were TNF, toll-like receptor 4 (TLR4), receptor-interacting serine/threonine kinase 2 (RIPK2), interleukin 1 beta (IL1B), receptor-interacting serine/threonine kinase 1 (RIPK1), fas-associated via death domain (FADD), Janus kinase 2 (JAK2), wnt family member 5A (WNT5A), TNF receptor-associated factor 6 (TRAF6) and innate immune signal transduction adaptor (MYD88). The signature we obtain using GO consists of the ranked systemic processes (y-axis) and prioritized genes (x-axis) (Figure 7). The first most prioritized gene was TNF, a prevalent target for many approved drugs such as anti-TNF agents, while all other nine genes have been implicated in studies for drug targeting in RA (89, 90).

Functional analysis with BioInfoMiner using the Human Phenotype Ontology gave 32 priority genes (Table S4) and enrichment in terms containing arthralgia, skin nodule, abnormality of the immune system, among others (Table S5), as we can see in Figure 8. Overall, the systemic functional analysis with BioInfoMiner further confirmed the validity of the model at the semantic level, complementary to the mechanistic one. The top 10 priority genes using PHO terms are interleukin 12A (IL12A), Fas cell surface death receptor (FAS), NRAS proto-oncogene (NRAS) GTPase, signal transducer and activator of transcription 3 (STAT3), protein tyrosine phosphatase (PTPN22), non-receptor type 22, major histocompatibility complex, class II, DR beta 1 (HLA-DRB1), Janus kinase 2 (JAK2), interferon regulatory factor 5 (IRF5), signal transducer and activator of transcription 4 (STAT4), catenin beta 1 (CTNNB1). All of these genes have been considered as putative drug targets in RA.

### Topological analysis of the RA map as a complex network

We imported the RA map to Cytoscape 3 to perform network analysis. The RA network comprises 1225 nodes and 1471 interactions (Figure 9). The analysis using Network Analyzer, a built-in tool of Cytoscape, revealed that the RA network consists of 30 connected components. These connected components correspond to the connected subgraphs, i.e. parts of the graph in which any node is accessible from any other node by a path, with a core subgraph of 1106 nodes and 1379 reactions and 29 smaller ones.

Node degree is a characteristic of the nodes of a network that describes the number of adjacent nodes (nodes directly connected to them). In directed networks such as signalling networks where the reactions are oriented (i.e. from the ECM to the nucleus) we can distinguish two types of node degree: the in-degree, meaning the number of directed edges that have the node as target, and the out-degree that is the number of directed edges that have the node as source. Node degree is an individual characteristic for each node, but a degree distribution can be computed to assess the diversity of the whole network.

The majority of biological networks display scale-free properties (91), which means that they contain a few central nodes that are highly connected (hubs) and several other loosely connected peripheral nodes. These networks follow a power law. This function indicates that there is a high diversity of node degrees which is why we describe these networks as ‘scale-free’.

First, we performed the analysis considering the network as undirected to obtain the overall degree distribution (in and out) and then as directed to get the in-degree and out-degree distributions. All node degree distributions follow a power law, showing that the RA network is indeed a scale-free network (91) (Figure 10).

In Table 1, we can see some of the topological characteristics of the RA network, analysed in Cytoscape. Each node has an average of 2. 299 neighbours (nodes to which it is connected). We used the degree distribution to obtain the hubs of the RA network, and in Table 2, we display the top 10 hubs. The network diameter of the RA network that corresponds to the maximum length of shortest paths between two nodes is 24 suggesting that the signal starting from ligand–receptor complexes in the membrane reaches most of the network within 24 steps. The characteristic path length of the network that corresponds to the expected distance between two connected nodes is approximately 10, meaning that the response to a signal and its propagation can occur relatively rapidly.

## Discussion

Visual representation of complex pathways and biological processes involved in a disease allows clinical and life sciences researchers to explore relevant mechanisms, which are often intricate and intertwined. Standardized representation and formalization of knowledge in the form of disease maps create an interface to a broad range of bioinformatics and modelling workflows. We present here a state-of-the-art, large-scale molecular interaction map for RA, which is to our knowledge the first SBGN-compliant Process Description disease map. While other efforts, such as the Asthma map, follow the SBGN format, their approach is different as they use three levels of granularity and different SBGN representations for every layer of information. The Process Description level for Asthma map consists of a set of separate modules that correspond to an Activity Flow layer, while the RA map is a global Process Description disease map.

All the components and reactions are annotated using only RA and human-specific studies. The RA map is part of the Disease Maps Project, a large scale community effort to comprehensively represent mechanisms for various diseases (13, 14) (http://disease-maps.org/). The community fosters the exchange of good practices and promotes the use of standards for the development of disease maps. The standards of curation and graphical representation, as well as the extensive annotation in both human and machine-readable formats of the RA map, ensure transparency, reproducibility and reusability of its content.

**Table 1:** Example of simple topological parameters obtained with Network Analyzer for the RA network

**Topological parameters**	**Corresponding values**
Connected component	30
Network diameter	24
Characteristic path length	10.099
Average number of neighbours	2.299
Number of nodes	1225
Isolated nodes	4

**Table 2:** Top 10 hubs of the RA map

**RA map nodes**	**Node degree**	**Role**	**Reference**
**NFKB**	28	Implicated in RA and inflammation	(92, 93)
**Inflammation**	14	A major characteristic of RA	(1, 66)
**AKT**	13	Regulates apoptosis in RA	(94, 95)
**Cell chemotaxis/recruitment/infiltration**	13	Implicated in RA	(96, 97)
**JUN**	13	Implicated in RA	(98, 99)
**MAPK1**	12	Implicated in RA	(100, 101)
**RAC1,2**	12	Implicated in RA	(102, 103)
**Cell growth/survival**	11	Major characteristic of RA	(66, 85)
**Osteoclastogenesis**	10	Results in bone damage in RA	(74, 104)
**TP53**	9	Involved in the apoptosis pathway implicated in RA	(105, 106)

In 2010 the first RA map was published by Wu et al. They used exclusively high-throughput RA experiments (mRNA, miRNA) described in 28 studies combined with data available in the KEGG database. A total of 435 species (263 proteins, 58 genes, 48 RNAs, seven simple molecules, one ion, one antisense RNA, 47 complexes), 265 reactions and 10 phenotypes involved in RA were identified using this approach. We decided to follow a different approach as described in the methodology section, in an attempt to limit false positives, increase confidence by incorporating experts’ advice and promote the use of SBGN standards for representation to assure reusability of the map. The new RA map we present here includes information from 353 peer-reviewed publications, and it has a significantly bigger size, as it features 506 species, 446 reactions and 8 phenotypes. The species in the map are classified to 303 proteins, 61 complexes, 106 genes, 106 RNA entities, 2 ions and 7 simple molecules.

The RA map can also be used as an interactive knowledge base, using the platform MINERVA and serve as a template for overlaying multiple datasets. Visualization of experimental data could help highlight aspects of the affected biological process and make differences between experimental conditions more evident. Visualizing the results of differential expression analysis of three datasets of gene expression of RA synovial tissues showed enrichment in all cellular phenotypes but not in apoptosis. This finding is in line with the fact that fibroblasts, which constitute a large percentage of the RA synoviocytes, have an apoptosis-resistant phenotype (107, 108).

We performed functional analysis and gene prioritization using BioInfoMiner (16). The genes that rank higher in this analysis are associated with many systemic processes and are considered as hubs in the semantic network. Along with prioritization, a pharmacogenomic analysis is provided since the hubs proposed are considered as putative drug targets. The results of the analyses using GO and PHO terms revealed known RA players, most of which have been already used as drug targets demonstrating that the RA map comprises well-characterized factors and captures most of the relevant systemic processes implicated in the disease.

The RA map serves as a curated knowledge base, but it can also be analysed as a complex network. Topological analysis can reveal underlying structural features of the RA map like unconnected parts of the network, or important hubs (well-connected nodes) which are otherwise hard to perceive in large-scale networks. The topological analysis performed in this study revealed connected and unconnected parts of the network. This result reflects our fragmented knowledge on the one hand, but also the use of stringent criteria for the nodes included in the map: experimentally validated interactions in at least two published studies, use of data of strictly human origin and disease-specific.

Another reason that contributes to the limited wiring of some of the RA map components is the unavailability of known interactions for newly discovered factors for RA. However, we keep them present because the RA map also works as an encyclopaedia for the disease, even if some parts of the puzzle are still missing.

The topological analysis also assists in the understanding of significantly connected nodes (hubs), placing them in their functional context. The top ten hubs of the RA map as seen in Table 1 (NFKB, AKT, Inflammation, Cell chemotaxis/recruitment/infiltration, JUN, MAPK1, RAC1,2 Cell growth/Survival, Osteoclastogenesis, TP53) are well-characterized factors implicated in the disease. Not surprisingly, four of them (AKT, MAPK1, RAC1,2, TP53) were also characterized as hubs in the first RA map by Wu *et al*., based on high-throughput data.

## Conclusion

The RA map is the fruit of interdisciplinary collaborations between clinicians, biologists and bioinformaticians. The aim was to build not only a knowledge repository but a versatile tool that can be used for various purposes. The RA map can offer to experimental biologists and clinicians easy access to all molecular pathways implicated in the disease along with references and annotations, to bioinformaticians a template for disease-specific pathway enrichment of *omic* datasets and finally, to computational modellers a mechanistic scaffold for the inference of a computational model (5, 6, 109), providing an intermediate step between a conceptual and an executable model.

## Ethics approval and consent to participate

Not applicable.

## Consent for publication

Not applicable.

## Availability of data and material

The RA map is freely accessible at ramap.elixir-luxembourg.org

The original CellDesigner XML file of the whole map can be downloaded from MINERVA from the INFO section by clicking on the source file (third tab in the left panel of MINERVA website). Right clicking on the main screen also gives an option to export the visible content in three formats – SBML, CellDesigner SBML and SBGN-ML.

## Competing interests

The authors declare that they have no competing interests. G.D.K. is currently also an employee at Regeneron Pharmaceuticals Inc. and declares no conflict of interest regarding the content of this manuscript.

## Authors’ contributions

**AN** designed the study, **A.N., V.S.** and **G.D.K.** built the new RA map content, **V.S.** drew the map including the addition of annotations and performed literature mining and topological analysis. **G.D.K.** validated the content, **V.S.** and **M.V.** performed DEG in datasets, **M.V.** produced scripts for all graphs, **A.N.** and **E.P.** performed functional and systemic analysis with BioInfoMiner, **V.S.** and **A.M.** validated the SBGN Process Description format of the RA map, **M.O.** and **P.G.** set up the MINERVA online version of the RA map, **M.O.** and **V.S.** added annotations to reactions of the RA map, **V.S.** and **E.B.** produced IPA RA datasets, **E.P.T.** helped with the validation of functional analyses results and **A.N.** and **V.S.** wrote the manuscript, all authors read and suggested modifications, all authors read and approved the final manuscript. **Corresponding author:** Dr Anna Niarakis.

## Supplementary Material

ra-figS1_baaa017

ra-figS2_baaa017

ra-figS3_baaa017
