# Supplementary figures and images for: RA-map: building a state-of-the-art interactive knowledge base for rheumatoid arthritis
Source: Database (Oxford). 2020 Apr 20;2020:baaa017. doi: 10.1093/database/baaa017 (PMC7170216; doi:10.1093/database/baaa017)

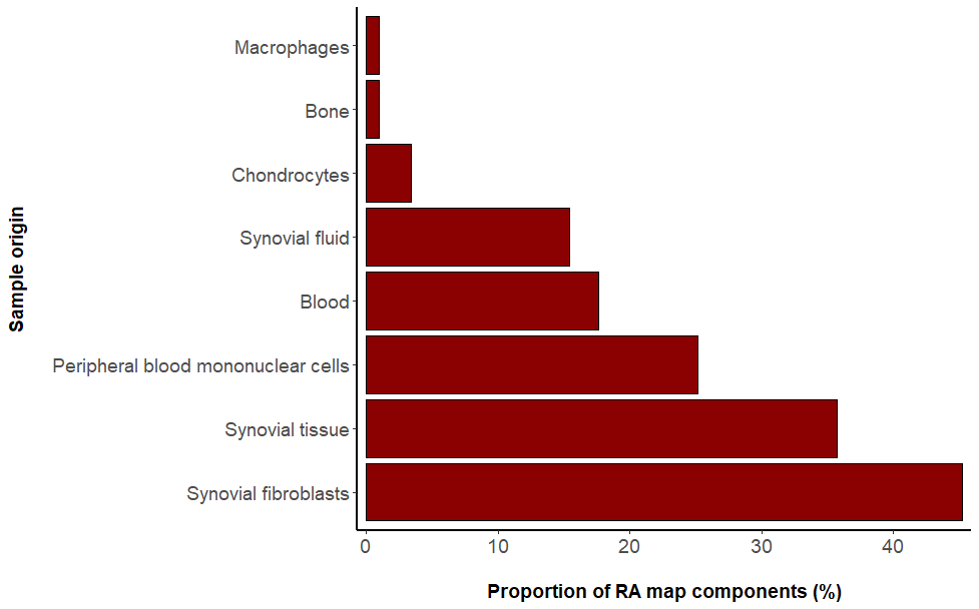

Supplement: ra-figS1_baaa017 [file ra-figs1_baaa017.png]

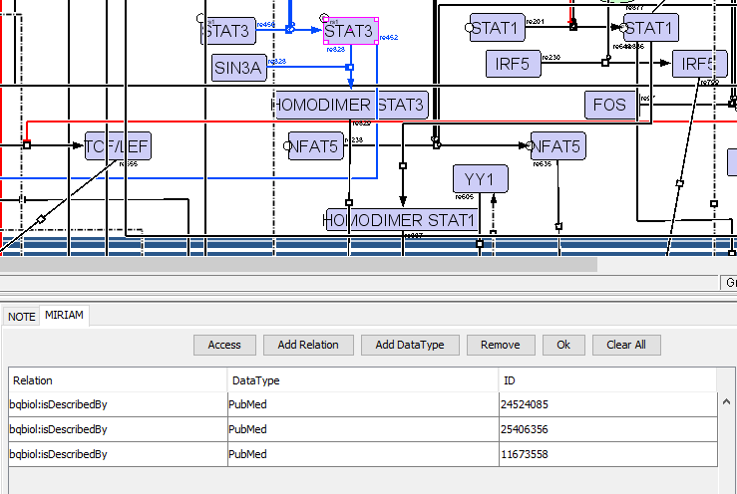

Supplement: ra-figS2_baaa017 [file ra-figs2_baaa017.png]

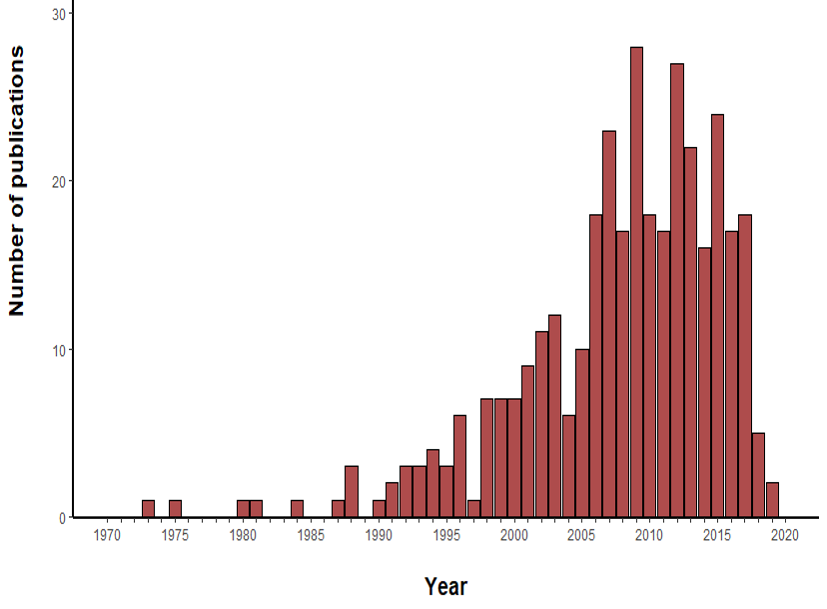

Supplement: ra-figS3_baaa017 [file ra-figs3_baaa017.png]
